# Prevalence and genetic diversity of noroviruses in adults with acute gastroenteritis in Huzhou, China, 2013–2014

**DOI:** 10.1007/s00705-015-2440-0

**Published:** 2015-05-08

**Authors:** Xiaofang Wu, Jiankang Han, Liping Chen, Deshun Xu, Yuehua Shen, Yunfeng Zha, Xiaojuan Zhu, Lei Ji

**Affiliations:** Huzhou Center for Disease Control and Prevention, 999 Changxing Road, Huzhou, 313000 Zhejiang China

## Abstract

Norovirus (NoV) infection is the most common cause of nonbacterial acute gastroenteritis, which affects both adults and children. However, the molecular epidemiology of NoV in adults with acute gastroenteritis in China has not been investigated extensively. In this study, we investigated the occurrence of NoV infections and analyzed the genetic diversity of NoV in adults with acute gastroenteritis in Huzhou, China. A total of 796 fecal samples were collected from outpatients (≥16 years of age) between March 2013 and February 2014. Real-time RT-PCR was performed to detect NoV genogroups I (GI) and II (GII). For genotyping, the capsid and RNA-dependent RNA polymerase (RdRp) genes were partially amplified and sequenced for phylogenetic analysis. NoVs were detected in 26.51 % (211/796) of the specimens, with GII being predominant, representing 96.20 % of the NoV infections. At least nine genotypes were identified among GI and GII specimens, including GI.P2/GI.2, GI.P3/GI.3, GI.P4/GI.4, GII.Pe/GII.4 Sydney_2012, GII.P12/GII.3, GII.P7/GII.6, GII.P16/GII.13, GII.Pe, and GII.Pg (RdRp only). This is the first report of a GII.P16/GII.13 recombinant virus in adults in China. GII.Pe/GII.4 Sydney_2012 was the most prevalent genotype and the only GII.4 variant identified during the study period. Our findings suggested that NoV was a common causative agent of acute gastroenteritis in adults in Huzhou, China. During the study period, the NoVs circulating in adults in Huzhou were predominantly GII.4 Sydney_2012 variants and GII NoV recombinants.

## Introduction

Acute gastroenteritis is one of the most common illnesses and a major public health problem worldwide. Since the development and application of novel sensitive molecular assays, noroviruses (NoVs) have been recognized as the leading cause of epidemics of gastroenteritis and an important cause of sporadic gastroenteritis in individuals of all ages in both developed and developing countries [4]. It is estimated that NoVs account for 12 % of severe gastroenteritis cases (hospitalized) among children less than 5 years old and 12 % of mild and moderate diarrhea cases (outpatient) among persons of all ages [26].

NoVs belong to the genus *Norovirus* in the family *Caliciviridae*. The viral genome is a single positive-strand RNA of 7.7 kb that contains three open reading frames (ORFs) [8]. ORF1 encodes several nonstructural proteins involved in replication of the genome, including RNA-dependent RNA polymerase (RdRp), nucleoside triphosphatases (NTPases), and proteases. ORF2 and ORF3 encode the major capsid protein VP1 and minor capsid protein VP2, respectively [31]. Due to the lack of a suitable cell-culture system for human NoV, genetic analysis is the principal method used to classify NoV strains. NoVs are a group of genetically diverse viruses that can be classified into six major phylogenetic clades, referred to as genogroups (GI to GVI) [21, 25, 43]. Genogroups are further classified into several genotypes. Although more than 30 genotypes within GI, GII, and GIV can infect humans, the majority of NoV-related outbreaks and sporadic cases of acute gastroenteritis are caused by a sub-genogroup of GII.4 strains [15, 44]. During the past decade, new variants of GII.4 strains have emerged every 2–3 years and have replaced the previously predominant GII.4 strains [30]. Emergence of these new NoV strains has often, but not always, been associated with increases in the number of outbreaks. RNA recombination is one of the major driving forces of virus evolution, and recombination of NoV genomes increases their genetic divergence. Analysis of these recombinants has suggested that the majority of recombination points are located near or within the ORF1/ORF2 overlap [2, 3].

Acute gastroenteritis is one of the most common public health problems in both China and other countries. During the past several years, most studies of NoV infection in China focused on the role of human NoV in acute gastroenteritis in children [9, 11, 42]. Although recent studies of NoV infection have focused on adults, no detailed examination of the genotype distribution among various age groups and according to season has been published [7, 12, 33, 38]. The present study was carried out to investigate the prevalence and genetic diversity of NoVs in adults with acute gastroenteritis in Huzhou, a medium-sized city located in eastern China.

## Materials and methods

### Study population and specimen collection

This study was conducted at the First People’s Hospital in Huzhou as part of the regional NoV gastroenteritis surveillance program. During March 2013 to February 2014, a total of 796 fecal specimens were collected from outpatients (≥16 years of age) with acute gastroenteritis. Acute gastroenteritis patients were defined as patients with diarrhea (three or more loose stools within 24 hours), which may be accompanied by vomiting, abdominal pain, fever, and nausea. All stool samples were freshly collected and sent to Huzhou Center for Disease Control and Prevention for routine NoV detection. Human ethics committee approval was not requested for this study, as all investigations were carried out on NoV strains; no human experimentation was conducted. The data are associated with NoV strains. No patient demographic information—other than age—was included in the analysis.

### Viral RNA extraction

Viral RNA was extracted from 200 μL of 10 % stool supernatant in MEM medium (Sigma-Aldrich, USA) using a QIAamp Viral RNA Mini Kit (QIAGEN, Hilden, Germany) according to the manufacturer’s instructions. The extracted RNA was dissolved in 60 μL of RNase-free water and stored at -70 °C until used.

### Norovirus detection

Genogroup-specific primers and probes described by Jothikumar *et al*. were used to detect NoVs by real-time RT-PCR [13]. The primer and probe sets JJV1F/JJV1R/JJV1P and JJV2F/COG2R/RING2-TP were used to screen for GI and GII NoV strains, respectively. Real-time RT-PCR was carried out using a One-Step PrimeScript^®^ RT-PCR Kit (DRR064) (TaKaRa, Dalian, China); the amplification conditions were described previously [10].

### Genomic amplification for genotyping

For genotyping, the primer sets G1SKF/G1SKR and G2SKF/G2SKR were used to amplify the 5′ end of the capsid gene (region C in ORF2) for GI and GII, respectively [14]. The primer set JV12Y/JV13I was used to amplify the 3′ end of the RdRp gene (region A in ORF1) [37]. For potential recombinant NoV strains (capsid and RdRp of a distinct genotype), a fragment covering the ORF1/ORF2 overlap was amplified using the primers JV12Y and G1SKR/G2SKR. A TaKaRa One-Step RT-PCR Kit (TaKaRa, Dalian, China) was used for amplification of region C and region A, and direct sequencing of PCR products was carried out by TaKaRa Biotechnology (Dalian, China). RT-PCR conditions were as described previously [10].

### Sequence analysis and molecular genotyping

All nucleotide sequences of RdRp and capsid were initially genotyped using a publicly accessible typing tool (http://www.rivm.nl/mpf/norovirus/typingtool) [16]. Phylogenetic analysis was performed using MEGA 5.2 software to confirm the results [32]. The phylogenetic tree was generated using the neighbor-joining method (distance calculation by the Kimura two-parameter correction, pairwise deletion), validated by 1000 bootstrap replicates. SimPlot (v. 3.5) analysis was used to identify putative recombination points and confirm putative recombinant strains [18]. The strains were designated by the genotype of RdRp followed by that of the capsid gene, according to the NoroNet [17].

### Nucleotide sequence accession numbers

GenBank accession numbers for the representative nucleotide sequences obtained in our study are KM462609–KM462657 and KM501034–KM501038.

## Results

### Norovirus infections

**Fig. 1:**
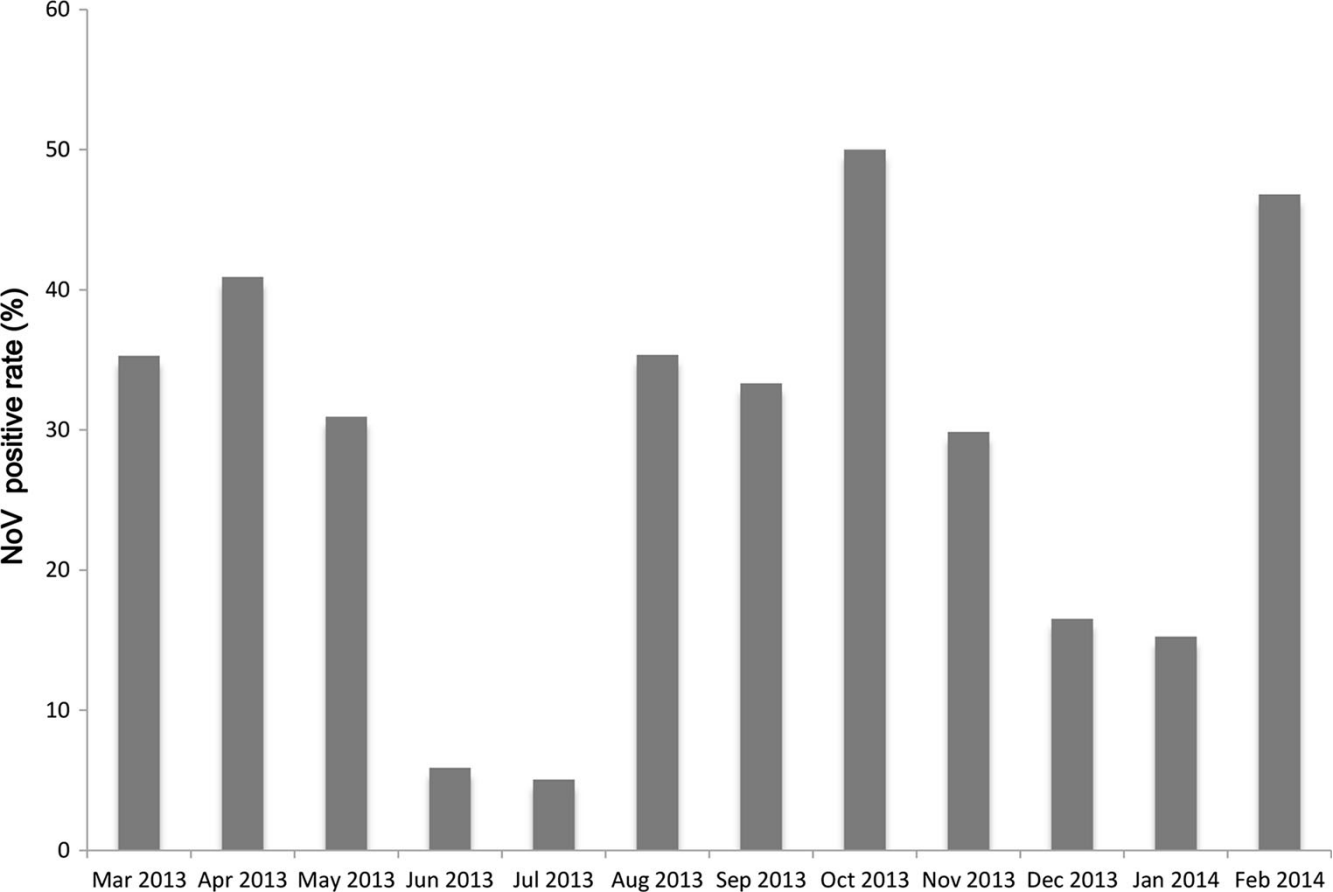
Monthly distribution of norovirus infections from March 2013 to February 2014. The highest detection rate (50.0 %) was observed in October 2013, and the lowest (5.1 %) in July 2013 (Mar, 35.3 %; Apr, 40.9 %; May, 30.9 %; Jun, 5.8 %; Jul, 5.1 %; Aug, 35.3 %; Sep, 33.3 %; Oct, 50.0 %; Nov, 29.8 %; Dec, 16.5 %; Jan, 15.2 %; Feb, 26.5 %)

From March 2013 to February 2014, 796 stool specimens collected from adult outpatients with acute gastroenteritis were tested for NoVs by real-time RT-PCR. Of the 796 specimens, 211 (26.51 %) were positive for NoV. Among the 211 NoV-positive specimens, 203 (96.20 %) viruses belonged to GII and eight (3.79 %) to GI. NoV infection was found in all age groups tested (16–20, 21–30, 31–40, 41–50, 51–60, and >60 years). The highest detection rate was in the 21–30-year age group (59/211, 27.9 %), followed by 51–60 years (44/211, 20.8 %), 41–50 years (35/211, 16.6 %), >60 years (32/211, 15.2 %), 31–40 years (26/211, 12.3 %) and 16–20 years (15/211, 7.1 %). The monthly distribution of the positive samples revealed that NoV infection could occur throughout the year (Fig. 1). The prevalence of NoV infection was higher from March to May (spring) and August to November (autumn). The prevalence of NoV infection was lower during the hottest months of the year (June and July).

### Genotypes

**Fig. 2:**
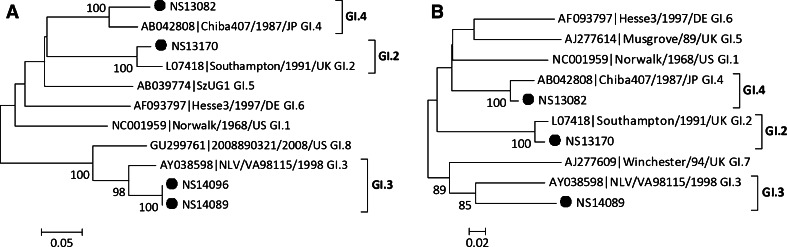
Phylogenetic analysis based on partial RdRp gene (A) and capsid gene (B) sequences of GI NoVs. NoV strains identified in this study are indicated by closed circles. The phylogenetic tree was generated using the neighbor-joining method, validated by 1000 bootstrap replicates. Bootstrap values ≥80 % are shown on the branches

**Fig. 3:**
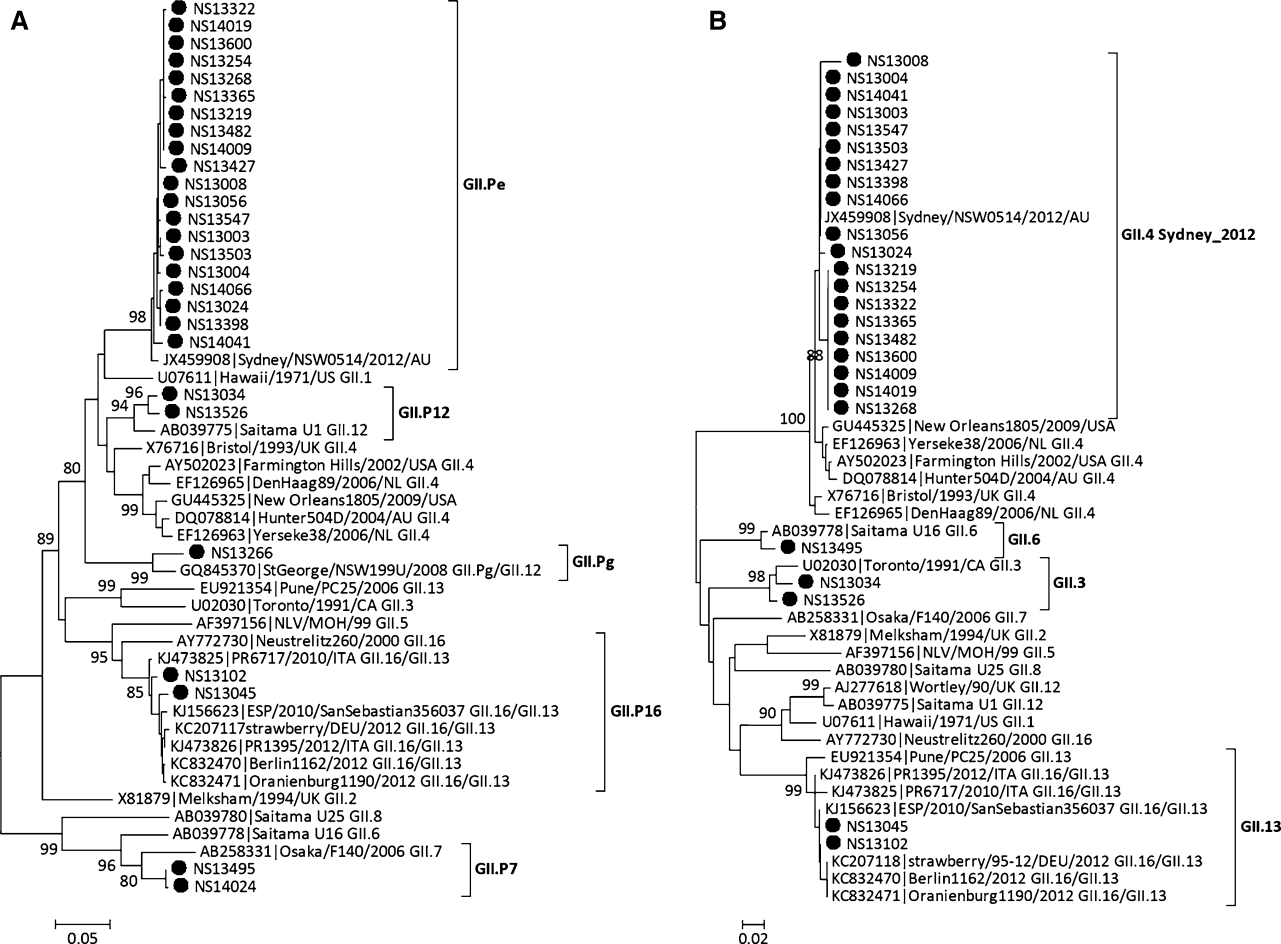
Phylogenetic analysis based on partial RdRp gene (A) and capsid gene (B) sequences of GII NoVs. NoV strains identified in this study are indicated by closed circles. The phylogenetic tree was generated using the neighbor-joining method, validated by 1000 bootstrap replicates. Bootstrap values ≥80 % are shown on the branches

Partial nucleotide sequences of two genes (capsid and RdRp) were obtained for 68 strains, and of the RdRp gene alone for 25 strains (Table 1). Based on RdRp and capsid sequences, the 68 NoV strains were clustered into seven genotypes: GI.P2/GI.2 (n = 1, 1.5 %), GI.P3/GI.3 (n = 1, 1.5 %), GI.P4/GI.4 (n = 1, 1.5 %), GII.Pe/GII.4 Sydney_2012 (n = 60, 88.2 %), GII.P7/GII.6 (n = 1, 1.5 %), GII.P12/GII.3 (n = 2, 2.9 %), and GII.P16/GII.13 (n = 2, 2.9 %). GII.Pe/GII.4 Sydney_2012 was the dominant genotype. For the 25 strains for which the sequence of only the RdRp gene was available, GII.Pe was the dominant genotype (n = 22, 88.0 %). GI.P3 (n = 1), GII.P7 (n = 1), and GII.Pg (n = 1) were also detected. A representative phylogenetic tree based on partial nucleotide sequences of the RdRp and capsid genes was generated by the neighbor-joining method (Figs. 2 and 3).

**Table 1:** Genotype distribution of identified NoV strains

Genogroup	Genotype		Genotype	
	RdRp/capsid	n (%)	RdRp	n (%)
GI	GI.P2/GI.2	1 (1.5 %)	GI.P3	1 (4.0 %)
	GI.P3/GI.3	1 (1.5 %)		
	GI.P4/GI.4	1 (1.5 %)		
GII	GII.Pe/GII.4 Sydney_2012	60 (88.2 %)	GII.Pe	22 (88.0 %)
	GII.P7/GII.6	1 (1.5 %)	GII.Pg	1 (4.0 %)
	GII.P12/GII.3	2 (2.9 %)	GII.P7	1 (4.0 %)
	GII.P16/GII.13	2 (2.9 %)		
Total		68		25

### Seasonal and age distribution of NoV genotypes

**Fig. 4:**
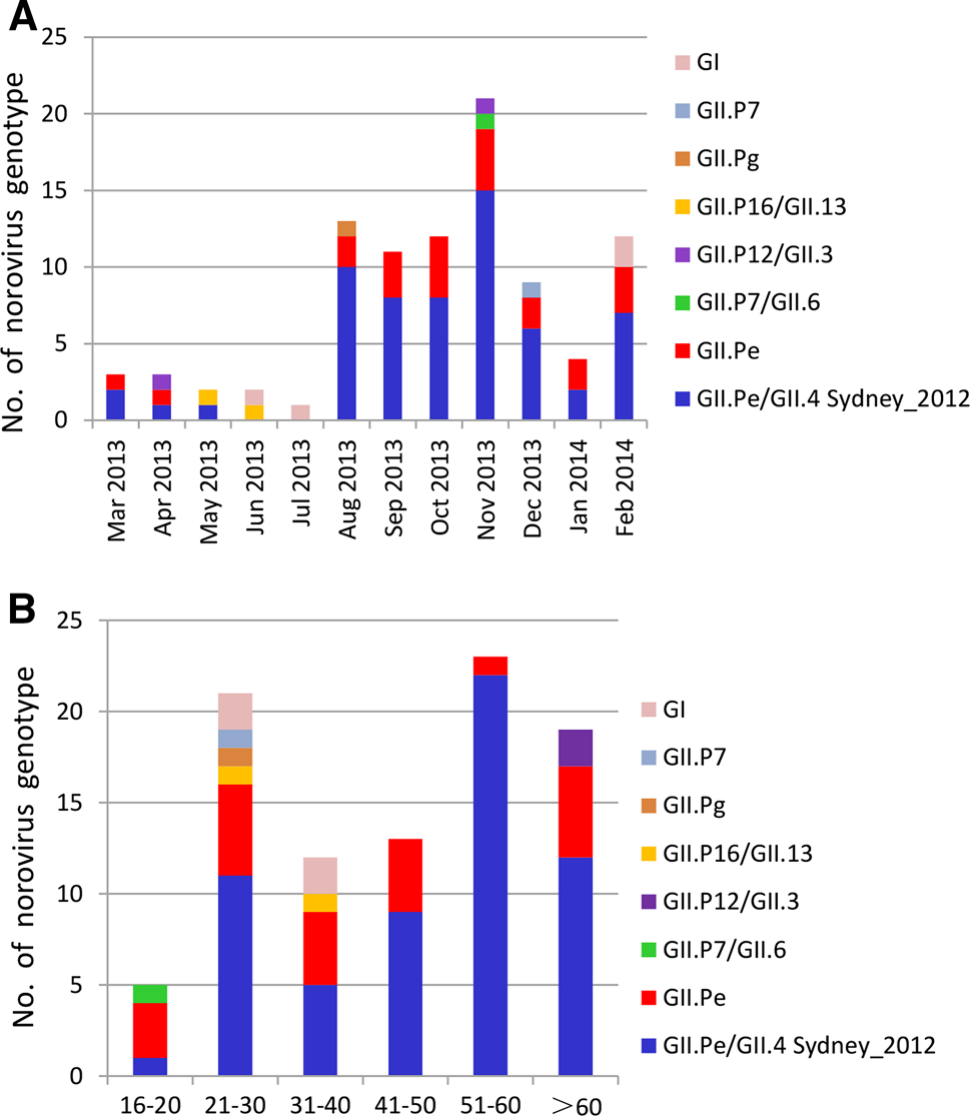
Distribution of NoV genotypes detected according to month (A) and age group (B)

During the study period, the dominant genotype GII.Pe/GII.4 Sydney_2012 was detected in almost all months, with the exceptions of June and July. The prevalence of NoV infection was lower during this period (Fig. 4A). The distribution of Nov genotypes according to age group is shown in Fig. 4B. For the GII.Pe/GII.4 Sydney_2012 strains, 33.7 % (22/60) were detected in 51–60-year age group, followed by >60 years (12/60, 20.0 %), 20–30 years (11/60, 18.3 %), 41–50 years (9/60, 15.0 %), 31–40 years (5/60, 8.3 %), and 16–20 years (1/60, 1.6 %).

### Identification of novel NoV recombinants

Genotype inconsistency between the RdRp and capsid regions was found in 65 strains, including the most prevalent type, GII.Pe/GII.4 Sydney_2012 (n = 60), followed by GII.P12/GII.3 (n = 2), GII.P16/GII.13 (n = 2), and GII.P7/GII.6 (n = 1). Recombinant strains of GII.P12/GII.3 (NS13034, NS13526), GII.P16/GII.13 (NS13045, NS13102), and GII.P7/GII.6 (NS13495) were detected for the first time in Huzhou.

**Fig. 5:**
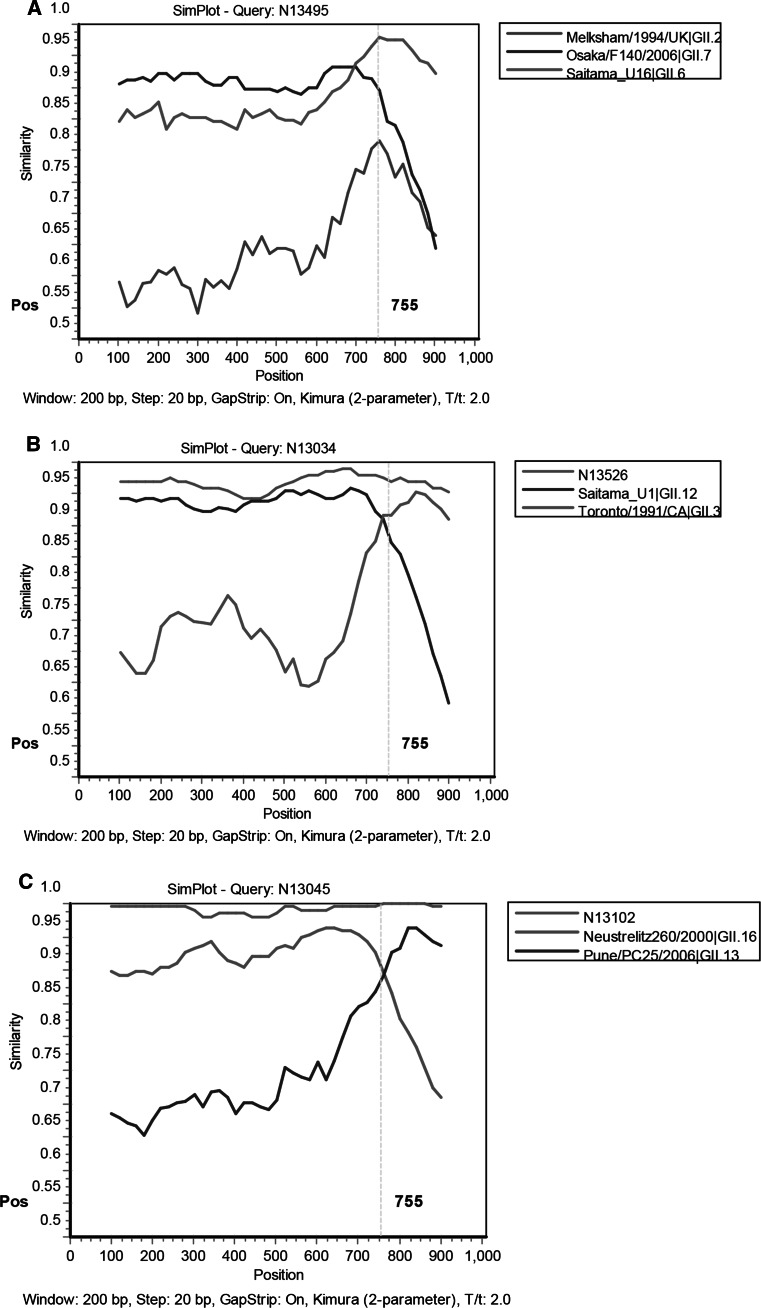
Simplot analysis for identification of recombinants detected in Huzhou. The vertical dashed line marks the beginning of the ORF1 region. (A) Simplot analysis of NS13495 using GII.2 (Melksham/1994/UK), GII.7 (Osaka/F140/2006), and GII.6 (Saitama_U16) as subtype references. (B) Simplot analysis of NS13034 using GII.12 (Saitama_U1) and GII.3 (Toronto/1991/CA) as subtype references; NS13526 served as the control strain. (C) Simplot analysis of NS13045 using GII.16 (Neustrelitz260/2000) and GII.13 (Pune/PC25/2006) as subtype references; NS13102 served as the control strain

Both to exclude the possibility that two NoV strains of different genotypes co-infected the same patient and to map the recombination site, a fragment (1066 bp) covering the ORF1/ORF2 overlap of these novel recombinant strains was amplified and analyzed using the SimPlot software. SimPlot analyses indicated potential recombination points for all recombinant types to be located near or within the ORF1/ORF2 junction (Fig. 5). The sequences of the five recombinant strains are available in GenBank under accession numbers KM501034 for GII.P7/GII.6, KM501035–KM501036 for GII.P12/GII.3, and KM501037–KM501038 for the GII.P16/GII.13 NoVs.

## Discussion

In this study, a systematic investigation of NoV infection was carried out in adult outpatients with acute gastroenteritis in Huzhou, China. The overall prevalence of NoV infection was 26.51 %. GII NoV infections were predominant, representing 96.20 % of the total NoV infections. This result is consistent with studies from other regions of China and in other countries. The prevalence of NoV in adults in the present study was higher than those reported previously from China (8.75–13.4 %), in which NoV was detected by conventional RT-PCR [7, 27, 28]. NoV infection was detected throughout the year. The peak rates of NoV infection occurred in autumn and spring, with the highest detection rate in October and April. However, according to existing data from multiple geographical areas of China, NoVs usually become highly active in mid-autumn and winter [7, 11, 28, 38, 42]. Whether the seasonal pattern of NoV is the same every year merits further investigation.

Analysis of the genetic diversity of the NoV strains in our study showed extensive co-circulation of various genotypes between March 2013 and February 2014 in Huzhou. A variety of GI and GII genotypes were identified, including GI.P2/GI.2, GI.P3/GI.3, GI.P4/GI.4, GII.Pe/GII.4 Sydney_2012, GII.P12/GII.3, GII.P7/GII.6, GII.P16/GII.13, GII.Pe, and GII.Pg (RdRp only). As reported previously, the GII.e polymerase genes can associate with several different capsid sequences; indeed, GII.Pe recombinants are frequently reported as the second most prevalent NoVs after GII.4 strains [6, 23]. In our study, GII.e polymerase genes were detected in 88.8 % (82/93) of the NoV strains based on RdRp sequence analysis. This high GII.Pe detection rate was most likely due to the emergence of the GII.4 variant Sydney_2012. To our knowledge, this new capsid variant is found most frequently in combination with the GII.Pe polymerase, but occasionally with the GII.4 New Orleans_2009 polymerase [22].

Although more than 30 genotypes have been identified, the majority of both sporadic NoV infections and outbreaks thereof are caused by GII.4 strains. Beginning in 1995, the emergence of novel GII.4 variants caused six pandemics of NoV-associated acute gastroenteritis, for example, in 2007–2009 (Den Haag 2006b variant) [34], 2009–2012 (New Orleans_2009 variant) [36], and most recently the Sydney_2012 variant. After the first detection of the Sydney_2012 variant in March 2012 in Australia, many countries, including China, reported increased levels of NoV activity associated with this novel variant during winter 2012–2013 [20, 29, 35]. We also reported similar patterns of circulating GII.4 variants in our previous study of the molecular epidemiology of NoV-associated acute gastroenteritis outbreaks in Huzhou between 2008 and 2012. GII.4 2006b was the only GII.4 variant circulating in 2008 and 2009 in the Huzhou area. The GII.4 New Orleans_2009 variant had been in co-circulation with the GII.4 2006b variant from 2010 to 2011 and accounted for 75 % of the typed outbreaks, and the newly reported GII.4 Sydney_2012 variant was first identified in November 2012 in Huzhou and caused two outbreaks in November 2012 [10]. Our present study revealed that GII.Pe/GII.4 Sydney_2012 was the only GII.4 variant detected in NoV-associated sporadic gastroenteritis in adults and was the most prevalent genotype in Huzhou from 2013 to 2014, suggesting the rapid spread of this variant in the population. Additionally, the GII.4 detection rate was high in elderly group of patients; indeed, 56.7 % (34/60) of GII.Pe/GII.4 Sydney_2012 cases were detected in the >50-year age group. This finding is consistent with a recent study conducted in adults in Beijing, China. The authors reported that the elderly group (>60 years of age) appeared to be more susceptible to GII.4 than to the other genotypes, and they speculated that the weak immune systems of elderly people and the persistence of the GII.4 genotype in human populations might explain this observation [33].

Recombination between NoV strains has occurred in nature at high frequency and represents a major driving force of viral evolution. Recombination allows the virus to increase its genetic fitness, to evolve, and to spread in the host population by escaping the host immune response [5]. Indeed, the vast majority of contemporary non-GII.4 NoVs are recombinant viruses, and some are characterized as globally prevalent NoV strains, such as GII.Pb/GII.3, GII.Pb/GII.13, and GII.Pg/GII.12 [39]. In the present study, recombinant strains represented an important portion, and 65 of the 68 (95.5 %) NoVs genotyped using both the RdRp and capsid genes corresponded to GII recombinant strains, highlighting the role of recombination in NoV evolution. Besides GII.Pe/GII.4 Sydney_2012, GII.P12/GII.3, GII.P7/GII.6, and GII.P16/GII.13 were detected for the first time in Huzhou. GII.12/GII.3 recombinant has been prevalent in China for several years, and infections with this variant were reported to be mainly in children [9, 28, 38]. GII.P7/GII.6 has also been detected in sporadic cases of acute gastroenteritis in Shanghai and Beijing, China [40, 41]. In 2013, we detected two rare recombinant strains, GII.P16/GII.13; this recombinant genotype has, to our knowledge, not been reported previously in China. In autumn 2012, a large-scale NoV gastroenteritis outbreak affecting about 11,000 people occurred in Germany, and epidemiological investigations suggested that frozen strawberries imported from China were the source of the outbreak. Genotyping of NoVs derived from the strawberries and patients revealed three distinct genotypes, including a GII.P16/GII.13 recombinant [19]. This was the first report of this NoV recombinant in Europe. In fact, recent studies indicated that GII.P16/GII.13 NoVs were already circulating in Spain and Italy in 2010, 2 years before the large-scale German outbreak [1, 24]. Based on phylogenetic analysis, Huzhou GII.P16/GII.13 strains (NS13045 and NS13102) were clustered with the GII.P16/GII.13 reference strains isolated from these European countries, and they shared 95.3–98.6 % and 98.3–99.6 % nucleotide sequence identity with these strains in segments of the RdRp and capsid genes, respectively. Detection of new NoV recombinant strains shortly after their initial detection in other countries suggests that some recombinant NoV strains can spread widely and rapidly.

In summary, our study provides a detailed description of the genetic diversity of NoVs in adults with acute gastroenteritis in Huzhou, China. During the study period, the NoVs circulating in adults in Huzhou were predominantly GII.4 Sydney_2012 variants and GII NoV recombinants. Three recombinant genotypes (GII.P12/GII.3, GII.P7/GII.6, and GII.P16/GII.13) were identified in this study by phylogenetic and Simplot analyses, among which the GII.P16/GII.13 recombinant was detected for the first time in adults in China. The findings of our study indicate that recombination makes an important contribution to the generation of diversity within NoVs.
